# Association Between Disability and Suicide Mortality in the Spanish Community-Dwelling Adult Population. A Population-Based Follow-Up Study

**DOI:** 10.3389/ijph.2024.1607344

**Published:** 2024-10-07

**Authors:** Roberto Pastor-Barriuso, Alicia Padrón-Monedero, Javier Almazán-Isla, Fernando J. García López, Jesús de Pedro-Cuesta, Javier Damián

**Affiliations:** ^1^ Carlos III Health Institute (ISCIII), Madrid, Spain; ^2^ Consortium for Biomedical Research in Epidemiology and Public Health, Madrid, Spain; ^3^ Consortium for Biomedical Research in Neurodegenerative Diseases, Madrid, Spain

**Keywords:** disability, suicide mortality, sociodemographic factors, follow-up study, sex differences

## Abstract

**Objectives:**

To assess the association of disability with suicide mortality, separately for women and men by age group.

**Methods:**

Information was obtained from a representative national sample of 161,809 community-dwelling adults (≥18 years). Participants contributed to follow-up time from baseline interview (2008) until suicide, death by other causes, or 2017. We calculated, by sex, standardized suicide mortality differences (SSMD), comparing persons with and without disabilities standardized to sociodemographic distribution of disability population.

**Results:**

29 women died by suicide during 800,754 person-years follow-up and 97 men during 735,709 person-years. Among women with disabilities, SSMD (95% Confidence interval) per 100,000 person-years at 5 years was 54.4 deaths (−17.2 to 126.1) [100.0 (−27.4 to 227.4) in women <65 years and −4.8 (−27.3 to 17.7) in women ≥65 years (P homogeneity = 0.11)]. Among men, SSMD increased by 122.2 deaths (4.1 to 240.3) [37.2 (−40.2 to 114.6) in men <65 years and 74.7 (−51.8 to 200.5) in men ≥65 years (P homogeneity = 0.62)].

**Conclusion:**

Suicide risk was higher in women and men with disability. In women higher risk was only notable for those <65. Men presented similar effects in both age groups. Nevertheless, due to imprecision of estimates, results should be viewed cautiously.

## Introduction

Suicide is an important public health problem [1–3] and a priority for the World Health Organization (WHO) [1]. Approximately 703,000 deaths by suicide occur each year, and in 2019 they represented 1.3% of global deaths [1]. Moreover, suicide has great importance in young populations because, in people aged 15–29 years, it constitutes the fourth leading cause of death globally and the second in Spain [1, 2]. Additionally, suicide is preventable [1] and the WHO recommends that suicide prevention strategies should be adapted to each country, by identifying vulnerable groups in each specific context [4]. In this regard, it has been suggested that having a disability could be a potential risk factor for suicide [5].

The concept of disability includes “impairments, activity limitations, and participation restrictions” [6] arising from the interaction between a health condition and the contextual factors of the individual [6], and it is considered to have a prevalence of 11.8% in higher income countries and 18.0% in lower income countries [6]. People with disabilities have an increased risk of depression [6, 7], anxiety [8], sleep problems [8], worse socioeconomic status [6], discrimination [6], isolation/solitude [8], and feelings of burdensomeness [9]. Some of these conditions may be related to an increased risk of suicide [4] and differ by sociodemographic groups [6].

A number of publications have specifically studied the relation between experiencing a disability and suicide [7, 9–16]. However, some of these were not representative of the general population and/or did not control for important confounders [13, 14, 16]. A limited number of population-based follow-up studies, have analyzed the likelihood of suicide in people with global assessments of disability, by sex and age groups [7, 10].

We previously studied the relation between disability and all-cause mortality in a representative sample of the adult population in Spain, finding a positive association [17]. In that work, we also showed a positive effect of disability on suicide mortality, among a group of 89 other causes of death. However, we consider it important to carry out a specific analysis that goes deeper into this relation, especially focusing on possible differences by sex and age groups, since the rates appear to be very different. In the present work, carried out in the same sample, the analyses will include effects measures that take into account the competing risks and additional co-variables. In addition, standardizations will be computed using the distribution of co-variables of the disabled population, which will provide a more approximate causal interpretation.

Thus, the objective of this research is to assess, in a representative sample of the adult population in Spain, the association of disability with suicide mortality, separately for women and men, globally and for the different groups of age and other sociodemographic factors.

## Methods

### Study Population

This cohort study included participants in the Spanish Survey on Disabilities, Personal Autonomy, and Dependency (*Encuesta Sobre Discapacidades, Autonomía Personal y Situaciones de Dependencia*, EDAD-08) [18] of the Spanish Statistical Office (Instituto Nacional de Estadística - INE). Participants were screened for baseline disability from November 2007 to February 2008 and were subsequently followed up for mortality through to December 2017. Participants were selected through a two-stage sampling stratified by province and municipality size. A total of 3,843 census tracts were initially selected with probability proportional to their size, and then 25 households were randomly sampled within each selected tract. Of the 84,497 eligible households, 63,541 agreed to participate (response rate of 75.2%). In addition, 27,749 ineligible or non-responding households were randomly substituted with other households in the same census tract. All 258,187 residents in the 91,290 participating households were screened for disability. Sampling weights were assigned to survey participants to account for the different selection probabilities by province and household composition and the distinct response rates by sex and age [18].

For the present study, we excluded 50,658 survey participants (19.6%) who lacked identifying data for mortality follow-up, 45,148 subjects (17.5%) under 18, and 572 participants (0.2%) with missing information on baseline sociodemographic characteristics. Thus, the final cohort included 161,809 adults (83,830 women and 77,979 men).

The Institute of Health Carlos III Research Ethics Committee approved the study (number CEI PI 17_2020).

### Baseline Disability and Mortality During Follow-Up

Baseline information about the disabilities of each household resident was obtained through personal interviews, first with the main household informant and then with those residents identified as having a possible disability. In the EDAD-08 survey, disability was defined as any important limitation to carrying out basic activities, which was caused by an impairment and had lasted or was expected to last more than 1 year. A disability was considered present even if it had been overcome with the use of external devices, or with the help or supervision of another person. The disability questionnaire included 44 items, grouped into the following eight domains: vision, hearing, communication, learning and application of knowledge and performance of tasks, mobility, self-care, home life, and interactions and interpersonal relationships. People who answered affirmatively to any of these questions formed the disability group. Further details on the disability assessment can be found elsewhere [18, 19].

Sex, age groups (18–34, 35–44, 45–54, 55–64, 65–74, 75–84, or ≥85 years), living with a partner (yes or no), educational level (less than primary, primary [6–11 years old], secondary [12–15], pre-university [16–17], or university [≥18]), and monthly household income (<1,000, 1,000–1,500, 1,500–2,000, 2,000–2,500, or ≥2,500 euros) were obtained by interview. For 7,881 of 81,282 households (9.7%) with missing income, we assigned the most frequent income category within their census tract. Place of residence was classified according to municipality size (<10,000, 10,000–20,000, 20,000–50,000, 50,000–100,000, or ≥100,000 inhabitants) and first-level nomenclature of territorial units (NUTS) [20] region (Northwest, Northeast, Madrid, Central, East, South, or Canary Islands).

Mortality data were provided by the Spanish Statistical Office (INE), since in Spain is mandatory by law that all deaths and their underlying causes “must be recorded in the Civil Register of the municipality where the death occurred” and “in the INE Central services, the files obtained from the recording are contrasted with those for recording deaths taken from the Civil Registers that are computerised and supplied to the INE by the General Directorate of Registries and Notaries of the Ministry of Justice” [21]. Suicide deaths corresponded to codes X60–X84 of the International Statistical Classification of Diseases and Related Health Problems, 10th Revision. Participants contributed follow-up time from their 2007–2008 baseline interview until suicide death, death from all other causes (competing risk), or 31 December 2017 (administrative censoring).

### Statistical Analysis

Due to strong differences in suicide mortality by sex, analyses were performed separately for women and men. The cumulative suicide mortality risk for disabled and non-disabled people was standardized to the weighted distribution of baseline sociodemographic characteristics in the community-dwelling disabled adult population of Spain by using marginal structural models with standardized-mortality-ratio weights [22]. We first fitted a sampling-weighted logistic regression model to estimate each participant’s population odds of being disabled, conditional on their observed sociodemographic characteristics, including age, living with partner, educational level, household income, municipality size, and geographical region. Standardization weights were set at one for disabled participants and were calculated as the above conditional odds of disability for non-disabled participants, further divided by the sampling-weighted marginal disability odds to stabilize weights across disability groups [22]. Combined weights were then assigned to survey participants as the product of sampling weights and standardization weights, thus correcting for selection bias and confounding by sociodemographic characteristics [23]. The mean (range) combined weights were 1.01 (0.01–76.3) for women and 1.02 (0.01–85.1) for men (Supplementary Figure S1). This weighting provided proper standardization, since the fully weighted distributions of baseline sociodemographic characteristics were similar between disabled and non-disabled people (Supplementary Table S1).

We obtained nonparametric and smooth estimates of the standardized cumulative suicide mortality curves in disabled and non-disabled people by using Kaplan-Meier methods [24], and spline-based survival models [25] weighted by the above combined weights and accounting for competing deaths from all other causes. For models based on splines, disability-specific log cumulative hazards were parameterized as distinct natural cubic splines of log time with a single internal knot at the 50th percentile [25, 26] which produced similar but more parsimonious cumulative mortality curves than nonparametric methods. We used spline-based survival models to estimate standardized differences and ratios in cumulative suicide mortality at 5 and 10 years of follow-up across disability groups accounting for other competing causes of death [24]. The 95% confidence intervals (CIs) were obtained by applying delta methods to robust standard errors of spline coefficients.

We fitted subgroup-specific weighted spline-based survival models accounting for other competing causes of death to evaluate potential heterogeneity in risk differences across baseline subgroups defined by age (18–64 or ≥65 years), living with a partner, educational level (primary or less, or secondary or more), household income (<1,500 or ≥1,500 euros), municipality size (<20,000 or ≥20,000 inhabitants), and region (North/Madrid/Central or East/South/Canary Islands). We used subgroup-specific combined weights to standardize cumulative suicide mortality to the weighted distribution of sociodemographic characteristics in the disabled population of each subgroup. Standardized differences in 5-year cumulative suicide mortality and 95% CIs between disabled and non-disabled people were estimated within each subgroup and tested for heterogeneity by using Wald tests. Statistical analyses were performed using the *stcompet*, *stpm2*, and *stpm2cif* commands in Stata, version 17 (StataCorp LP, College Station, Texas 77845 United States) and graphics were produced in R, version 4 (R Foundation for Statistical Computing, Vienna, Austria).

## Results

In the community-dwelling adult population of Spain, the prevalence of disability (95% CI) was 12.9% (12.6% to 13.1%) in women and 9.0% (8.7% to 9.2%) in men. Women with disability were older, had lower educational level and household income, and were more likely to live alone in small municipalities in the central and southern regions of Spain than women without disability. Similar but less pronounced sociodemographic differences were observed between men with and without disability (Table 1).

**TABLE 1:** Baseline sociodemographic characteristics of participants by sex and disability in the Survey on Disabilities, Personal Autonomy, and Dependency, Spain, 2007–2008^a^.

Characteristic	Women			*P*-value^b^	Men			*P*-value^b^
	Overall	Non-disabled people	Disabled people		Overall	Non-disabled people	Disabled people	
No. of participants	83,830 (100)	72,648 (87.1)	11,182 (12.9)		77,979 (100)	70,618 (91.0)	7,361 (9.0)	
Age (years)				<0.001				<0.001
18–34	20,042 (26.7)	19,638 (30.1)	404 (4.0)		20,274 (29.9)	19,746 (31.9)	528 (8.6)	
35–44	16,789 (19.7)	16,112 (21.7)	677 (6.0)		15,914 (21.0)	15,266 (22.1)	648 (10.1)	
45–54	14,842 (16.8)	13,690 (17.8)	1,152 (10.4)		14,356 (17.3)	13,420 (17.7)	936 (12.7)	
55–64	12,070 (13.8)	10,396 (13.6)	1,674 (14.9)		11,402 (13.6)	10,160 (13.4)	1,242 (16.3)	
65–74	10,021 (11.2)	7,728 (9.9)	2,293 (19.9)		8,827 (10.1)	7,374 (9.2)	1,453 (19.3)	
75–84	7,682 (8.8)	4,365 (5.8)	3,317 (29.3)		5,943 (6.6)	4,105 (5.0)	1,838 (23.0)	
≥85	2,384 (2.9)	719 (1.0)	1,665 (15.5)		1,263 (1.5)	547 (0.7)	716 (9.9)	
Living with partner				<0.001				0.03
Yes	53,895 (63.1)	48,362 (65.4)	5,533 (47.3)		53,703 (66.4)	48,670 (66.5)	5,033 (65.0)	
No	29,935 (36.9)	24,286 (34.6)	5,649 (52.7)		24,276 (33.6)	21,948 (33.5)	2,328 (35.0)	
Educational level				<0.001				<0.001
Less than primary	16,273 (18.4)	10,774 (13.8)	5,499 (49.0)		11,816 (13.7)	8,971 (11.4)	2,845 (37.1)	
Primary	22,651 (24.9)	19,296 (24.3)	3,355 (29.0)		21,800 (25.3)	19,439 (24.8)	2,361 (30.7)	
Secondary	11,289 (13.3)	10,308 (14.0)	981 (8.8)		11,987 (15.3)	11,211 (15.7)	776 (10.9)	
Pre-university	16,516 (21.1)	15,758 (23.1)	758 (7.3)		16,463 (22.7)	15,691 (23.8)	772 (11.9)	
University	17,101 (22.3)	16,512 (24.7)	589 (5.8)		15,913 (23.0)	15,306 (24.4)	607 (9.5)	
Monthly household income (euros)				<0.001				<0.001
<1,000	23,358 (25.4)	17,776 (22.0)	5,582 (48.6)		18,494 (21.0)	15,247 (19.0)	3,247 (41.8)	
1,000–1,500	20,741 (23.8)	18,090 (23.9)	2,651 (22.9)		19,822 (24.4)	17,924 (24.3)	1898 (25.6)	
1,500–2,000	15,159 (18.5)	13,758 (19.3)	1,401 (13.3)		15,010 (19.7)	13,954 (20.1)	1,056 (15.2)	
2,000–2,500	10,099 (12.9)	9,398 (13.8)	701 (6.5)		10,030 (13.7)	9,457 (14.3)	573 (8.2)	
≥2,500	14,473 (19.4)	13,626 (21.0)	847 (8.7)		14,623 (21.1)	14,036 (22.3)	587 (9.2)	
Municipality size (inhabitants)				<0.001				<0.001
<10,000	22,477 (20.4)	19,077 (19.8)	3,400 (24.1)		22,820 (22.2)	20,380 (21.8)	2,440 (25.6)	
10,000–20,000	9,079 (10.3)	7,906 (10.3)	1,173 (10.1)		8,651 (10.7)	7,841 (10.7)	810 (10.7)	
20,000–50,000	10,833 (14.7)	9,570 (14.9)	1,263 (12.7)		10,069 (14.8)	9,234 (14.9)	835 (13.5)	
50,000–100,000	6,967 (10.3)	6,158 (10.6)	809 (8.6)		6,282 (10.1)	5,765 (10.3)	517 (8.3)	
≥100,000	34,474 (44.4)	29,937 (44.3)	4,537 (44.5)		30,157 (42.2)	27,398 (42.2)	2,759 (41.9)	
Geographical region				<0.001				<0.001
Northwest	9,046 (10.3)	7,642 (10.1)	1,404 (11.6)		8,264 (9.9)	7,354 (9.8)	910 (10.9)	
Northeast	12,403 (10.5)	11,079 (10.7)	1,324 (9.4)		11,721 (10.4)	10,797 (10.5)	924 (9.4)	
Madrid	5,095 (14.0)	4,569 (14.4)	526 (11.4)		4,613 (13.7)	4,262 (13.9)	351 (11.8)	
Central	17,998 (12.5)	15,375 (12.1)	2,623 (15.0)		17,272 (12.7)	15,459 (12.5)	1813 (14.8)	
East	15,322 (28.9)	13,385 (29.2)	1,937 (27.1)		14,065 (29.1)	12,814 (29.2)	1,251 (27.5)	
South	21,007 (20.0)	17,955 (19.6)	3,052 (22.4)		19,371 (20.3)	17,476 (20.1)	1,895 (22.1)	
Canary Islands	2,959 (3.9)	2,643 (4.0)	316 (3.0)		2,673 (3.9)	2,456 (3.9)	217 (3.5)	

Association between disability and suicide mortality in the Spanish community-dwelling adult population. A population-based follow-up study, Spain, 2007–2017.

^a^ Unweighted counts (sampling-weighted percentages).

^b^ *P*-value for homogeneity of sampling-weighted percentages between disabled and non-disabled adults.

**FIGURE 1:**
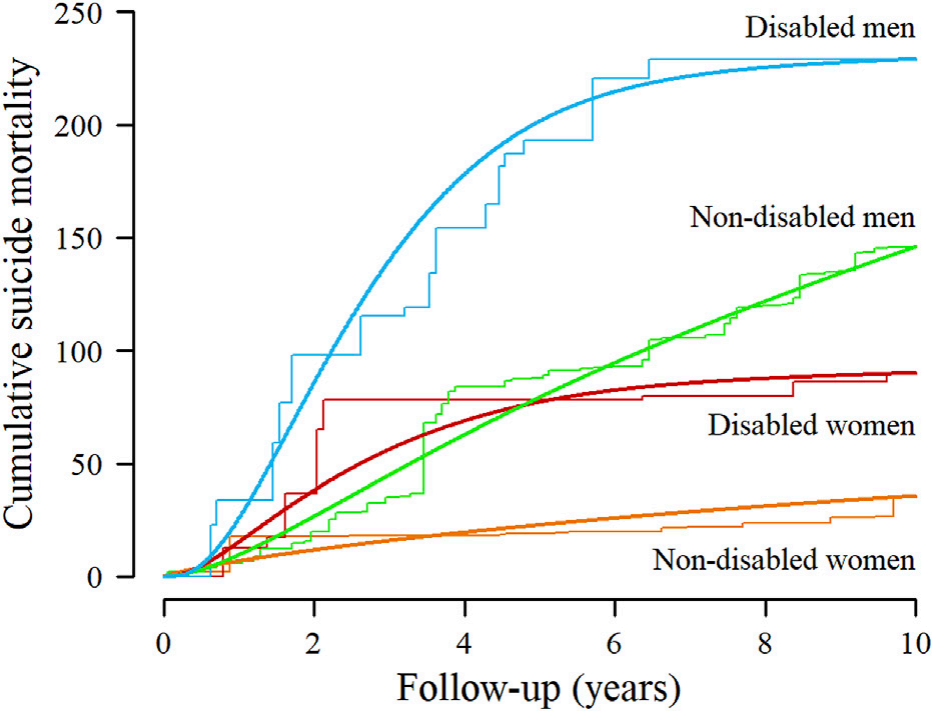
Standardized cumulative suicide mortality per 100,000 people by sex and disability among community-dwelling adults in Spain, 2007–2008 to 2017. Parametric cumulative suicide mortality curves (smooth lines) were estimated from spline-based survival models and nonparametric curves (step functions) from Kaplan-Meier methods, both stratified by sex and disability, weighted by combined weights, and accounting for competing deaths from other causes. Combined weights were used to standardize cumulative suicide mortality in disabled and non-disabled people to the sex-specific distribution of baseline sociodemographic characteristics in the community-dwelling disabled population, including age, living with partner, educational level, household income, municipality size, and geographical region. Association between disability and suicide mortality in the Spanish community-dwelling adult population. A population-based follow-up study, Spain, 2007–2017.

During 800,754 person-years of follow-up, 29 women died from suicide and 7,932 from other causes, corresponding to mortality rates of 3.6 and 952.0 deaths per 100,000 person-years. Among men, there were 97 deaths from suicide and 9,020 from other causes during 735,709 person-years of follow-up, with higher mortality rates of 11.6 and 1118.0 deaths per 100,000 person-years (Table 2 *)*. The unstandardized cumulative suicide mortality risks per 100,000, comparing disabled with non-disabled women and men, are provided in Table 2. After standardizing to the weighted distribution of baseline sociodemographic characteristics in the community-dwelling disabled population and accounting for competing deaths from other causes, suicide mortality was consistently higher at any follow-up time among disabled women and men in comparison with non-disabled (Figure 1). Compared with adults without a baseline disability, the standardized cumulative suicide mortality (95% CI) at 5 and 10 years of follow-up was 54.4 deaths (−17.2 to 126.1) and 54.4 deaths (−22.7 to 131.6) per 100,000 disabled women; and 122.2 deaths (4.1 to 240.3) and 83.1 deaths (−40.7 to 206.8) per 100,000 disabled men. Similarly, the standardized suicide mortality risk ratios (95% CIs) at 5 and 10 years of follow-up were 3.39 (0.61 to 18.66) and 2.53 (0.72–8.93) comparing disabled with non-disabled women, and 2.54 (1.22 to 5.27) and 1.57 (0.86 to 2.87) comparing disabled with non-disabled men (Table 2 *)*.

**TABLE 2:** Standardized differences and ratios in cumulative suicide mortality at 5 and 10 years of follow-up by disability, among community-dwelling adult women and men in Spain, 2007–2008 to 2017.

	Women		Men	
	Non-disabled people	Disabled people	Non-disabled people	Disabled people
No. of person-years	711,784	88,970	679,402	56,307
No. of deaths				
Suicide	20	9	77	20
All other causes	3,645	4,287	5,926	3,094
Mortality rate^a^				
Suicide	2.7	11.3	10.2	29.8
All other causes	485.7	4,853.4	784.7	5,380.9
5-year follow-up				
Cumulative suicide mortality^b^	10.5	78.1	46.9	193.1
Standardized mortality difference^c^ (95% CI)	0.0 (reference)	54.4 (−17.2 to 126.1)	0.0 (reference)	122.2 (4.1 to 240.3)
Standardized mortality ratio^c^ (95% CI)	1.00 (reference)	3.39 (0.61 to 18.66)	1.00 (reference)	2.54 (1.22 to 5.27)
10-year follow-up				
Cumulative suicide mortality^b^	26.8	90.1	98.7	229.1
Standardized mortality difference^c^ (95% CI)	0.0 (reference)	54.4 (−22.7 to 131.6)	0.0 (reference)	83.1 (−40.7 to 206.8)
Standardized mortality ratio^c^ (95% CI)	1.00 (reference)	2.53 (0.72 to 8.93)	1.00 (reference)	1.57 (0.86 to 2.87)

Association between disability and suicide mortality in the Spanish community-dwelling adult population. A population-based follow-up study, Spain, 2007–2017.

^a^ Sampling-weighted mortality rates per 100,000 person-years.

^b^ Unstandardized cumulative suicide mortality risks per 100,000 people at the specified follow-up times were obtained using sampling-weighted Kaplan-Meier methods stratified by sex and disability and accounting for competing deaths from other causes.

^c^ Standardized differences and ratios in cumulative suicide mortality at the specified follow-up times comparing disabled and non-disabled adults were obtained from spline-based survival models stratified by sex and disability, weighted by combined weights, and accounting for competing deaths from other causes, with 95% confidence intervals (CIs) derived by applying delta methods to robust standard errors of spline coefficients. Combined weights were used to standardize cumulative suicide mortality in disabled and non-disabled people to the sex-specific distribution of baseline sociodemographic characteristics in the community-dwelling disabled population, including age, living with partner, educational level, household income, municipality size, and geographical region.

**FIGURE 2:**
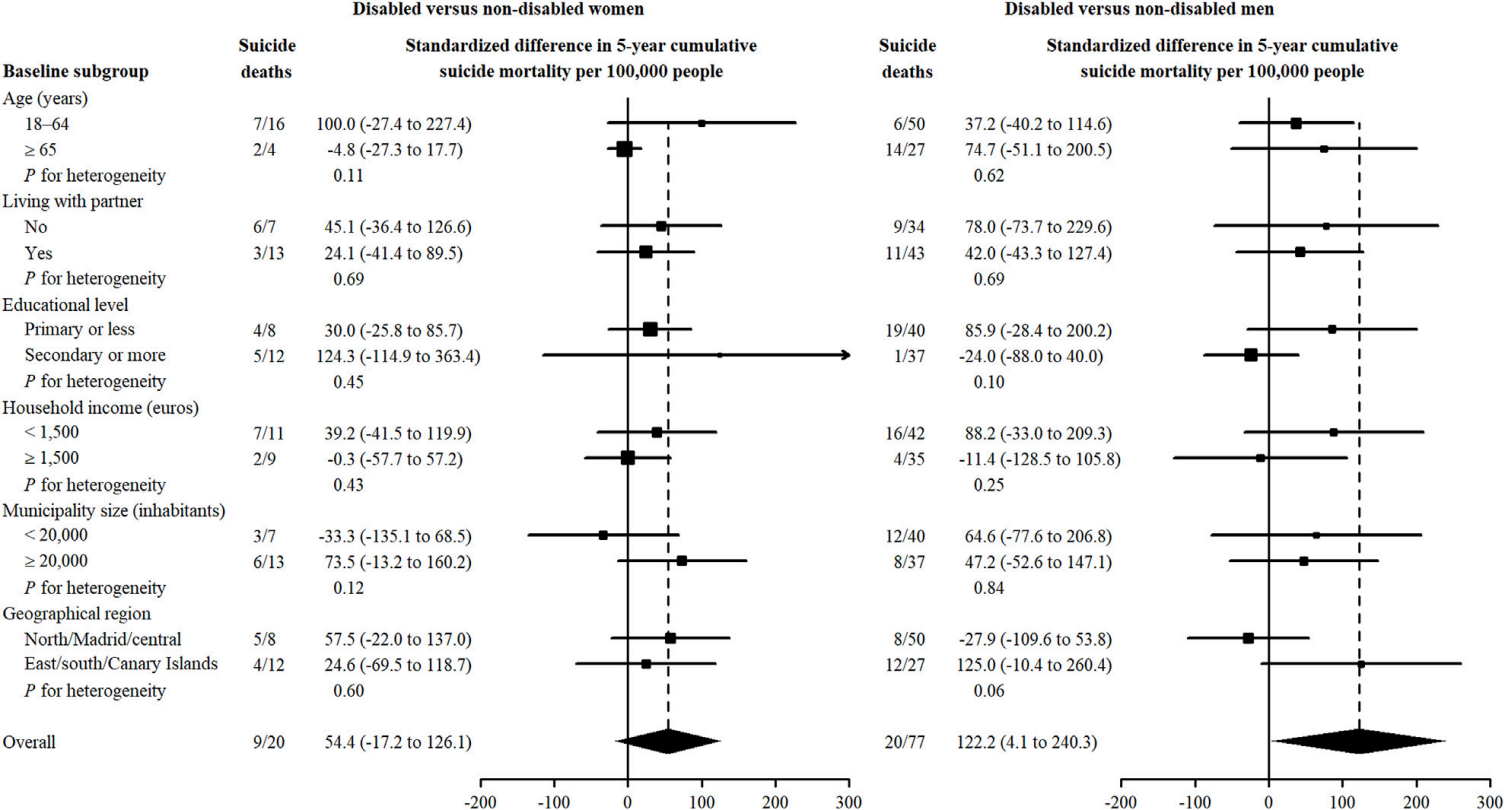
Standardized differences in 5-year cumulative suicide mortality comparing disabled with non-disabled women and men by subgroup of community-dwelling adults in Spain, 2007–2008 to 2017. Subgroup-specific risk differences (squares with area inversely proportional to the variance) and their 95% confidence intervals (horizontal lines) were obtained from spline-based survival models stratified by sex, covariate group, and disability, weighted by combined weights, and accounting for competing deaths from other causes. Subgroup-specific combined weights were used to standardize cumulative suicide mortality in disabled and non-disabled people to the distribution of baseline sociodemographic characteristics in the community-dwelling disabled population within each sex and covariate group, including age, living with partner, educational level, household income, municipality size, and geographical region. Association between disability and suicide mortality in the Spanish community-dwelling adult population. A population-based follow-up study, Spain, 2007–2017.

In subgroup analyses, the excess risk of suicide death associated with disability was larger among women younger than 65 years at baseline (*P* for homogeneity = 0.11) and those living in more populated areas (*P* = 0.12). The standardized 5-year suicide mortality risk (95% CI) was 100.0 deaths (−27.4 to 227.4) per 100,000 disabled women aged 18–64 years, and 73.5 deaths (−13.2 to 160.2) per 100,000 disabled women residing in municipalities larger than 20,000 inhabitants compared to women with no disability (Figure 2). In addition, disability was associated with more marked outcomes in suicide risk among less educated men (*P* for homogeneity = 0.10) and those living in the East and South regions (*P* = 0.06). The standardized 5-year suicide risk (95% CI) was 85.9 deaths (−28.4 to 200.2) per 100,000 disabled men with primary or lower educational level and 125.0 deaths (−10.4 to 260.4) per 100,000 disabled men residing in Eastern, Southern Spain, or the Canary Islands (Figure 2).

## Discussion

In this large, population-based follow-up study, the cumulative suicide mortality for both sexes was higher at any follow-up time among disabled people, compared to non-disabled people. Nevertheless, it remained relatively stable after the fifth year of follow-up. These results are consistent with studies reporting that, in persons with disability, time to death by suicide is skewed towards early points [7]. Additionally, the impact of disability appeared to differ by sex and age group. In women, the increased risk of suicide mortality was only noteworthy in those younger than 65. In men, the estimates were similar for both age groups. However, these results should be viewed with caution due to the imprecision of estimates. Further studies are needed to confirm these patterns.

A limited number of studies have assessed the association between disability and suicide mortality in nationally representative samples and, in general, our results are consistent with their findings. To the best of our knowledge, the works of Park et al. [10], and Lee et al. [7] are the only studies that analyzed the hazard ratio (HR) of suicide in people with any of the different types of disability, by sex and age groups in population-based follow-ups. Park et al. in an 11-year population-based follow-up study, found that men with disabilities had an adjusted risk of suicide 1.60 times higher than men without disabilities [10], which is roughly similar to our results. It is interesting to highlight that, in their study, this association was smaller for women (adjusted risk of suicide 1.26) [10]. Additionally, they found that the association between disability and risk of suicide was substantial only for those younger than 60 years old and increased when decreasing the age group [10] which is comparable to the results for severe disability found in other studies [11]. On the other hand, Lee et al., in a 10-year population-based follow-up study, reported that the adjusted HR for people with disability was 1.9 times higher compared to those without disability [7]. Moreover, in contrast to Park et al., the HR were very similar for both sexes [7]. They also found a decreased risk when increasing the age group, and suggested that the age at which the disability occurs is a more important risk factor for suicide than the current age [7]. Also, Onyeka et al. [11] in a 4-year population-based follow-up study, showed that adults with a disability were over 1.5 times more likely to die by suicide compared to those with no limitations, after adjustment for poor mental health. Moreover, and consistent with Park et al. [10] the relationship between severe disability and death by suicide was notable only for those under 60 years old [10, 11]. Along the same lines, Turvey et al., in a longitudinal cohort study of people 65 years and older, did not find increased odds ratios of suicide in those with functional impairment; however, due to small numbers, they did not provide estimates by sex [15]. It is relevant to highlight that neither of these studies analyzed this association for the different age groups by sex [7, 10, 11, 15], and our results suggest that the lower suicide risk for the older age group [7, 10, 15] could be attributable, at least partially, to the weight of estimates for women. Cao et al. [13] found that older people from rural China with a severe disability were 1.4 more likely to die by suicide, compared to those without disability. Kaplan et al. also found that people reporting a functional limitation (through an *ad hoc* question that did not included disability duration) had roughly 3 times higher suicide mortality risk [12] and, finally, Kim et al. [14] found that people with disability had 2.5 times higher mortality rates by suicide than the general population, which is consistent with our results for men.

Numerous potential underlying pathophysiological mechanisms for the relation between disability and suicide have been described. Most are related to psychiatric and sociodemographic factors that are more likely in people with disability (depression [5, 7, 8], worse socioeconomic conditions [6], discrimination [6], isolation/solitude [8], and feelings of burdensomeness [9]) and could be associated with an increased risk of suicide [4]. It is interesting to highlight, that the Interpersonal Theory of Suicide, hypothesizes that suicide ideation is fomented by the integration of perceived burdensomeness and thwarted belongingness (feeling not to be part of a social group) [27, 28]. Moreover, it is considered that the relationship between perceived burdensomeness, thwarted belongingness, and suicidal ideation is mediated by lack of meaning in life that underlies the concept of demoralization [28].

Thus, it is interesting to consider the information about the subjective experiences of these patients in relation to their disability. Constanza et al. in a quantitative-qualitative observational mixed method study analyzed information from validated quantitative questionnaires and qualitative open-ended questions of seventy participants at the Multidisciplinary Pain Center of the Geneva University Hospitals. They assessed that in those patients, social interactions and activities were related to meaning in life, and pain could impact on meaningful relationships and activities, the ability to enjoy and feeling pleasure, lack or loss of vital objectives and meaning, fear of the future, mood disturbances, a sense of loss and despair that induced deep moral suffering. More specifically, the patients refer that the disability puts them in the situation of reviewing life goals and expectations, that has an important impact in their meaning in life [29]. Thus, the disability undermines meaning in life, directly or by impairing its two domains (social interactions and activities) in a vicious circle related to suicidal ideation [29].

In any case, the analysis of disability associated with suicide mortality is complex, and, as already suggested, suicide mortality risk in people with disability may be disproportionately distributed in relation to the different types of disability [7, 10], severities of disability [10, 11], and sociodemographic groups [7, 10, 11]. Furthermore, some potentially disabling conditions may lead to psychiatric symptoms [30] and there appears to be a bidirectional relation between psychiatric symptoms and function [31]. On the other hand, it is very unlikely that diseases that may lead to disability (other than depression and psychiatric disorders) would increase suicide mortality risk directly, and not through mechanisms related to disability and/or psychiatric disorders and, thus, it is not very probable that they would act as confounding factors.

The analyses performed in the present study suggest some interesting patterns. Our estimates are relatively invariant across age groups for men; however, for women, they are only remarkable for those <65 years. Thus, it appears that disability-related suicide for older women is different than for younger women and/or men. As a possible explanation, it has been suggested that older people may anticipate activity limitations related to age and better manage their role expectations [6, 11] while younger people may experience such situations as a heavier burden not being able to perform the activities expected of a person their age [11]. According to our results, this possible explanation would apply mainly to women. Qualitative studies could be of interest to assess the subjective experience of these persons that may provide additional information to explain this pattern.

### Strengths and Limitations

A main strength of the study is the large nationally representative sample of the Spanish adult population and a long follow-up. Additionally, data were obtained from official statistics, and analyses were stratified by sex and adjusted for potential sociodemographic confounders. The study has several limitations, however. First, 20% of participants from the sample lacked mortality information. Nevertheless, we may assume that they were randomly distributed, since the missing information was due to problems in the identification of the participants from the beginning of the follow-up. Thus, we can reasonably believe that any potential bias in the results would have been small. Second, although we adjusted for numerous potential sociodemographic confounders, we cannot rule out that some of our estimates may suffer from some residual confounding. Third, the information provided by the individuals about their disability status was self-reported (although obtained by trained examiners/interviewers), thus we cannot discard some degree of both over- and under-reporting. Nevertheless, these potential measurement errors would have diluted the observed associations and led them towards the null. Fourth, we could not obtain mortality data for the institutionalized population. Since that population of older people may have different characteristics (including diverse and more severe disabilities, a different patient role in mental health services, and a different management of suicide attempts) we cannot assure that our results would apply to the institutionalized population. Fifth, some subgroup analyses had a small number of cases, resulting in imprecise estimates. Sixth, the definition of disability in the EDAD-08 survey did not make it possible to discriminate by the severity of disability, and we cannot rule out that different degrees of disability could be related to different suicide mortality risks for our global population and/or several subgroups. Seventh, the mortality data provided by the Spanish Statistical Office did not include a specific code for “sequels of intentionally self-inflicted injuries.” As they were included in the group of “events of undetermined intention” we could not obtain specific information of this outcome. That being said, the total number of deceased of the group “events of undetermined intention” included five people; consequently, the non-inclusion of potential deaths caused as a sequel of a self-inflicted injury would have had a negligible impact on our results. Eighth, we only assessed disability at the baseline survey, we cannot rule out that new disabilities (particularly severe disabilities) that may have appeared during the follow-up, could have had an impact in suicide risk.

Finally, our results could lead to considering that the population with disability should be evaluated by their practitioners regarding the convenience of undergoing a specific psychiatric evaluation (in a multidisciplinary way) taking into account their sociodemographic characteristics in addition to other relevant risk factors; as well as the enforcement of social and educational policies that provide support to this population.

### Conclusion

The risk of suicide appeared to be higher for both women and men with disability, although the association between disability and suicide seemed to differ by sex and age group. In women, the increased risk of suicide mortality was only noteworthy in those younger than 65 years old, while for men there were similar effects in both age groups. Nevertheless, these results should be viewed with caution due to the imprecision of estimates. Further studies are needed to confirm this pattern, in order to take into account these potential differences in subsequent research and in programs aimed at quantifying or reducing the apparent increased suicide risk in persons with disability.
