# Supplementary material for: Association Between Disability and Suicide Mortality in the Spanish Community-Dwelling Adult Population. A Population-Based Follow-Up Study
Source: Int J Public Health. 2024 Oct 7;69:1607344. doi: 10.3389/ijph.2024.1607344 (PMC11491392; doi:10.3389/ijph.2024.1607344)
Supplement: Supplementary file 1 [file DataSheet1.docx]

**Supplementary Table 1.** Distribution of baseline sociodemographic characteristics by disability after standardization to the community-dwelling disabled adult population of Spain, 2007–2008

|  | **Women** | |  | **Men** | |  |
| --- | --- | --- | --- | --- | --- | --- |
| **Characteristic** | **Non-disabled people*** | **Disabled people*** | ***P* value†** | **Non-disabled people*** | **Disabled people*** | ***P* value†** |
| Age (years) |  |  | 0.91 |  |  | 0.54 |
| 18–34 | 3.9 | 4.0 |  | 8.4 | 8.6 |  |
| 35–44 | 5.9 | 6.0 |  | 9.7 | 10.1 |  |
| 45–54 | 10.2 | 10.4 |  | 12.3 | 12.7 |  |
| 55–64 | 14.7 | 14.9 |  | 15.9 | 16.3 |  |
| 65–74 | 19.8 | 19.9 |  | 19.2 | 19.3 |  |
| 75–84 | 29.4 | 29.3 |  | 23.6 | 23.0 |  |
| ≥85 | 16.2 | 15.5 |  | 10.8 | 9.9 |  |
| Living with partner |  |  | 0.12 |  |  | 0.03 |
| No | 53.9 | 52.7 |  | 36.9 | 35.0 |  |
| Yes | 46.1 | 47.3 |  | 63.1 | 65.0 |  |
| Educational level |  |  | 0.50 |  |  | 0.19 |
| Less than primary | 50.2 | 49.0 |  | 39.0 | 37.1 |  |
| Primary | 28.2 | 29.0 |  | 29.9 | 30.7 |  |
| Secondary | 8.7 | 8.8 |  | 10.6 | 10.9 |  |
| Pre-university | 7.2 | 7.3 |  | 11.5 | 11.9 |  |
| University | 5.7 | 5.8 |  | 9.0 | 9.5 |  |
| Monthly household income (euros) | |  | 0.08 |  |  | 0.47 |
| <1,000 | 50.5 | 48.6 |  | 43.2 | 41.8 |  |
| 1,000–1,500 | 22.6 | 22.9 |  | 25.3 | 25.6 |  |
| 1,500–2,000 | 12.5 | 13.3 |  | 14.8 | 15.2 |  |
| 2,000–2,500 | 6.1 | 6.5 |  | 7.8 | 8.2 |  |
| ≥2,500 | 8.2 | 8.7 |  | 8.9 | 9.2 |  |
| Municipality size (inhabitants) | |  | 0.98 |  |  | 0.94 |
| <10,000 | 24.3 | 24.1 |  | 25.8 | 25.6 |  |
| 10,000–20,000 | 9.9 | 10.1 |  | 10.9 | 10.7 |  |
| 20,000–50,000 | 12.8 | 12.7 |  | 13.7 | 13.5 |  |
| 50,000–100,000 | 8.5 | 8.6 |  | 8.0 | 8.3 |  |
| ≥100,000 | 44.4 | 44.5 |  | 41.7 | 41.9 |  |
| Geographical region |  |  | 0.97 |  |  | 0.99 |
| Northwest | 11.3 | 11.6 |  | 10.9 | 10.9 |  |
| Northeast | 9.3 | 9.4 |  | 9.7 | 9.4 |  |
| Madrid | 11.3 | 11.4 |  | 11.7 | 11.8 |  |
| Central | 15.3 | 15.0 |  | 14.7 | 14.8 |  |
| East | 27.5 | 27.1 |  | 27.8 | 27.5 |  |
| South | 22.2 | 22.4 |  | 21.8 | 22.1 |  |
| Canary Islands | 3.1 | 3.0 |  | 3.4 | 3.5 |  |

* Fully weighted percentages taking into account both sampling and standardization weights.

† *P* value for homogeneity of fully weighted percentages between disabled and non-disabled adults.

Association between disability and suicide mortality in the Spanish community-dwelling adult population. A population-based follow-up study, Spain, 2007-2017.

**
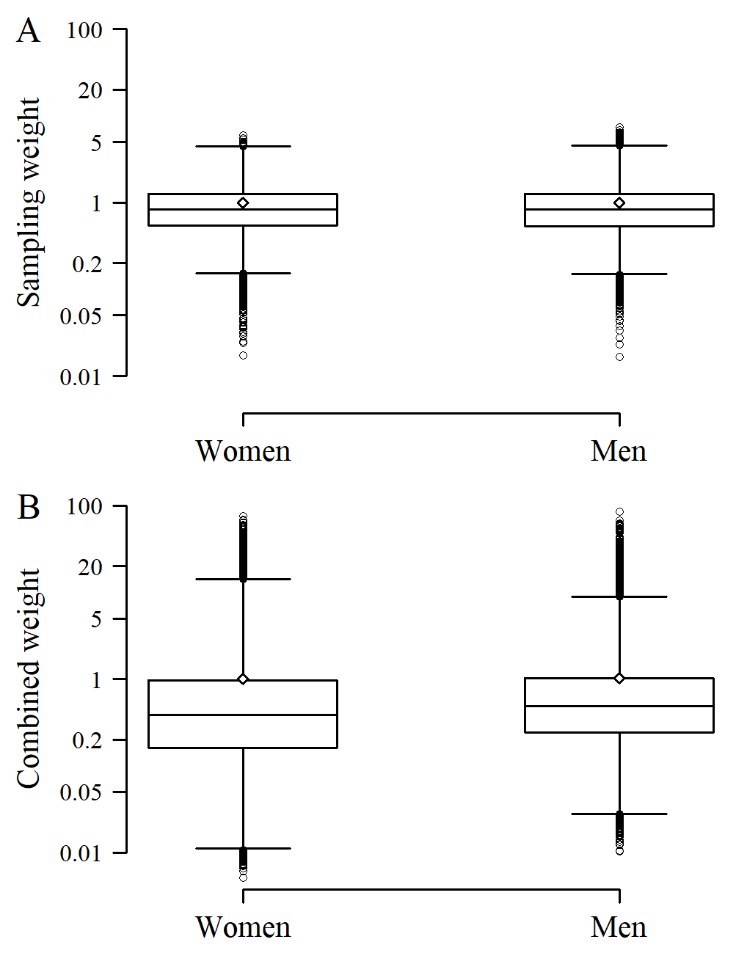
Supplementary Figure 1.** Distribution of sampling weights (A) and combined weights taking into account sampling and standardization (B) among women and men in the Survey on Disabilities, Personal Autonomy, and Dependency, Spain, 2007–2008.

Boxes represent the mean (diamond), median (middle horizontal line), quartiles (border horizontal lines), and individual outlying weights (circles).

Association between disability and suicide mortality in the Spanish community-dwelling adult population. A population-based follow-up study, Spain, 2007-2017.
